# Effect of TiO_2_-ZnO-MgO Mixed Oxide on Microbial Growth and Toxicity against *Artemia salina*

**DOI:** 10.3390/nano9070992

**Published:** 2019-07-10

**Authors:** Luis M. Anaya-Esparza, Napoleón González-Silva, Elhadi M. Yahia, O. A. González-Vargas, Efigenia Montalvo-González, Alejandro Pérez-Larios

**Affiliations:** 1 Laboratorio Integral de Investigación en Alimentos, Tecnológico Nacional de México/Instituto Tecnológico de Tepic, Av. Tecnológico 255 Fracc, Lagos del Country, Tepic 63175, Nayarit, Mexico; 2 División de Ciencias Agropecuarias e Ingenierías, Centro Universitario de los Altos, Universidad de Guadalajara, Av. Rafael Casillas Aceves 1200, Tepatitlán de Morelos 47600, Jalisco, Mexico; 3 Facultad de Ciencias Naturales, Universidad Autónoma de Querétaro, Avenida de las Ciencias S/N, Juriquilla, Santiago de Querétaro 76230, Querétaro, Mexico; 4 Departamento de Ingeniería en Control y Automatización, Escuela Superior de Ingeniería Mecánica y Eléctrica-Zacatenco, Instituto Politécnico Nacional, UPALM, Av. Politécnico S/N, Col. Zacatenco, Alcaldía Gustavo A. Madero, Ciudad de México 07738, Mexico

**Keywords:** mixed oxides, nanomaterials, antimicrobial activity, toxicity, *Artemia salina*

## Abstract

Mixed oxide nanoparticles (MONs, TiO_2_–ZnO–MgO) obtained by the sol-gel method were characterized by transmission electron microscopy, (TEM, HRTEM, and SAED) and thermogravimetric analysis (TGA/DTGA–DTA). Furthermore, the effect of MONs on microbial growth (growth profiling curve, lethal and sublethal effect) of *Escherichia coli*, *Salmonella paratyphi*, *Staphylococcus aureus* and *Listeria monocytogenes*, as well as the toxicity against *Artemia salina* by the lethal concentration test (LC_50_) were evaluated. MONs exhibited a near-spherical in shape, polycrystalline structure and mean sizes from 17 to 23 nm. The thermal analysis revealed that the anatase phase of MONs is completed around 480–500 °C. The normal growth of all bacteria tested is affected by the MONs presence compared with the control group. MONs also exhibited a reduction on the plate count from 0.58 to 2.10 log CFU/mL with a sublethal cell injury from 17 to 98%. No significant toxicity within 24 h was observed on *A. salina*. A bacteriostatic effect of MONs on bacteria was evidenced, which was strongly influenced by the type of bacteria, as well as no toxic effects (LC_50_ >1000 mg/L; TiO_2_–ZnO (5%)–MgO (5%)) on *A. salina* were detected. This study demonstrates the potential of MONs for industrial applications.

## 1. Introduction

Titanium dioxide (TiO_2_) is one of the most employed nanomaterials in a wide range of applications, including as white pigment for pharmaceutical and food industry, sunscreen for skin, photo-catalysts, water treatment, hydrogen production, ethylene scavenging and antimicrobial activity [1,2,3,4]. Therefore, its production and use are projected to increase by >500% by the year 2025 [5]. Furthermore, TiO_2_ is compatible with a large number of elements and compounds (e.g., ZnO, MgO and Ag) via doping or mixed oxide systems (binary or ternary systems) [6], enhancing their electric, physicochemical and mechanical properties (photocatalytic activity, large surface area and high pore volume) [1,3]. There are several pathways (hydrothermal, co-precipitation, combustion, vapor deposition, and sol-gel method) to synthesize TiO_2_ nanoparticles (alone or combined) [7,8,9,10]. Nonetheless, the sol-gel method is an appropriate, relatively simple, and economic technique to synthesize TiO_2_ and/or hybrid (inorganic–inorganic or organic–inorganic) nanomaterials in its mixed oxide form. This method involves the hydrolysis and condensation reactions on the precursors [6,11].

Antimicrobial activity is an interesting property of TiO_2_ and/or mixed oxide system (MONs) based TiO_2_, which have been tested against Gram–negative and Gram–positive bacteria [1,6,12]. TiO_2_ is widely used for antimicrobial effects due to its excellent photocatalytic properties and ability to produce reactive oxygen species (ROS) under ultraviolet light radiation (UVR, UV-lamp, fluorescent lamp, and solar energy). It can cause damage to bacteria cell membranes promoting cell lysis and cell shrinking [13,14]. The antibacterial activity of TiO_2_ has been enhanced by the presence of other materials, mainly due to its efficient electron-hole separation and ROS production [13]. Fu et al. [15] reported a major antibacterial activity of nanocomposite from Au–TiO_2_ compared to pure TiO_2_, and similar trends were also reported by Li et al. [16] using Ag–TiO_2_–chitosan against *Escherichia coli*, *Pseudomonas aeruginosa*, and *Staphylococcus aureus*. Likewise, Hassan et al. [17] reported a superior antimicrobial effect of Ce_2_O_3_–TiO_2_ compared to pure TiO_2_ against *S. aureus* and *Salmonella typhimurium*. Recently we have reported that MONs–based TiO_2_ (TiO_2x_–ZnO_x_–MgO_x_) exhibited major antimicrobial activity compared to pure TiO_2_ against *E. coli*, *S. paratyphi*, *S. aureus* and *Listeria monocytogenes* [6]. Although the antibacterial activity of TiO_2_ and MONs was previously investigated, the antimicrobial tests of inorganic nanoparticles are usually performed by agar disc diffusion assay or by optical density [1,6,12,18,19,20], reporting a significant inhibition of cell growth on Gram–negative and Gram–positive bacteria [14]. On the other hand, a bacteriostatic effect of TiO_2_ nanoparticles has been reported previously by Venkatasubbu et al. [18], who suggested that more than one antimicrobial activity test is needed to apply when inorganic nanoparticles are investigated, with the aim to elucidate if the particles exhibit a bacteriostatic or bactericidal effect.

Another important aspect for the potential use of TiO_2_ and MONs (based–TiO_2_) is their possible human health risk, particularly for the potential food and pharmaceutical applications [21,22,23]. *Artemia salina* (brine shrimp), is an important crustacean model used for the preliminary assessment of general toxicity of various compounds such as plant extracts, dental materials, fungal toxins, pesticides and inorganic nanoparticles (alone, doped or mixed oxide systems) [24]. In addition, *A. salina* can be used as nauplii (within the 24 h of life) or in the adult state (3 weeks of life, adult measure 2 cm in length) for the test [25]. The toxicity assay is based on the lethality of brine shrimps in the presence of any biological and/or chemical compounds [26]. This method provides a simple and inexpensive assay for the toxicity screening, with highly reproducible and accurate results since *A. salina* exhibits a short life cycle (4 weeks), and is adaptable to salinity and temperature [26]. Most of the available data on the toxicity of TiO_2_–based nanomaterials have been reported on models such as *A. salina* [24,25] and *Daphnia magna* [26]. Ates et al. [24] evaluated the effect of aqueous suspensions of TiO_2_ nanoparticles on *A. salina* and reported that neither nauplii nor adults showed non–toxic effects at 100 mg/L of TiO_2_ after 24 h of exposition. However, earlier studies have demonstrated that TiO_2_ under UVR can produce ROS species [27,28,29,30], but these negative effects were not observed at similar concentrations of TiO_2_ without exposure to UVR or under natural sunlight [26,29,30]. Furthermore, Gambardella [31] reported that some selected metal oxide (SnO_2_, CeO_2_, and Fe_3_O_4_) nanoparticles (20 to 30 nm) at 1 mg/mL did not induce significant mortality of *A. salina*. Nonetheless, it has been reported that TiO_2_ toxicity degree is less toxic than other inorganic nanoparticles (Ag > CuO > ZnO > Au > TiO_2_ > SiO_2_) [32]. Moreover, this status may be affected (positively or negatively) by the presence of other materials into the new nanocomposite. Ozkan et al. [33] reported that Ag–TiO_2_ nanocomposite exhibited major toxic effects on *A. salina* compared to the pure TiO_2_. On the other hand, Cortés et al. [34] indicated that C_5_H_8_O_2_ toxicity on *Artemia franciscana* can be diminished by its combination with NaClO. In this context, the toxic status of any ternary–mixed oxide system is limited.

In this work, we synthesized a ternary oxide system based TiO_2_ (TiO_2_–ZnO–MgO) by the sol–gel method and characterized it by transmission electron microscopy (TEM, HRTEM, and SAED) and thermogravimetric analysis (TGA/DTGA–DTA). In addition, we evaluated the growth curve profiling, the lethal and sublethal effect of TiO_2_–ZnO–MgO nanomaterial on *E. coli*, *S. paratyphi*, *S. aureus*, and *L. monocytogenes*, as well as their toxicity against *A. salina*.

## 2. Materials and Methods

### 2.1. Material Preparation

Materials (TiO_2_-ZnO-MgO) were prepared by the sol–gel method as previously described [6]. A measured quantity of ethanol (44 mL) and distilled water (18 mL) were mixed with the different precursor (zinc nitrate and magnesium di-ter-butoxide) amounts to obtain solids with 1–1, 3–3 and 5–5% in weight of Zn and Mg, respectively (Table 1). The solution was then adjusted to pH 3 using HNO_3_ 0.1 N. The solutions were heated under reflux at 70 °C and titanium (IV) butoxide (44 mL), and were then added dropwise and maintained during 24 h under magnetic stirring until the gels were formed. Gels formed were dried at 100 °C for 24 h to eliminate the ethanolic residues and the solids were ground, the dried powders were annealed at 500 °C for 5 h in a static air atmosphere (at a heating rate of 2 °C/min). A reference pure TiO_2_ sample was prepared in the same way described above but without the addition of the precursors (Zn–Mg). Reagents were purchased from Sigma-Aldrich Chemical Co. St. Louis, MO, USA.

### 2.2. Material Characterization

Details of physical and textural properties (specific surface area, pore diameter, pore volume, color attributes and band gap energy), including structural properties (X–ray diffraction and FT–IR studies), the superficial morphologies (scanning electron microscopy), as well as their elemental composition by energy dispersive spectroscopy (EDS) of materials employed in this study have been reported in earlier work [6]. The morphology, selected area diffraction pattern (SAED), lattice fringes (HRTEM) and size of the samples were determined using a field–emission gun transmission electron microscopy (Jeol microscope, JEM-ARM200F, Tokyo, Japan) operating at 200 kV (all TEM images were analyzed using Gatan Micrograph software v. 3.7.0, Gatan Inc., Pleasanton, CA, USA). The thermal stability of the samples was carried out by thermogravimetric and differential thermal analysis (TGA/DTGA–DTA), performed at 30 °C to 1000 °C in a thermogravimetric analyzer SDT Q600 V20.5 Build 15 (Eschborn, Germany) under air flow (100 mL/min) at a heating rate of 10 °C/min.

### 2.3. Bacterial Strains

Bacterial strains used for the evaluation of the effect of TiO_2_–ZnO–MgO mixed oxide on microbial growth were: *E. coli* (ATCC 8739), *S. paratyphi* (ATCC 9150), *S. aureus* (ATCC 33862) and *L. monocytogenes* (ATCC 15313), which are frequently occurring foodborne pathogenic microorganisms [1]. Microorganisms were cultivated in Lauria–Bertani (LB) medium [pancreatic digest of casein 10 g/L (Bacto^TM^ tryptone), yeast extract 5 g/L (BBL^TM^), sodium chloride 5 g/L (J.T. Baker^®^) at pH 7.2 ± 0.2] and incubated at 37 °C for 24 h [35] prior to analysis. Bacterial strains were purchased from Microbiologics^®^ (Saint Cloud, MN, USA) and rehydrated according to the instructions of the manufacturer. All materials and reagents (including MONs) used for the different microbial tests were previously sterilized at 121 °C for 15 min.

### 2.4. Antibacterial Assay

#### 2.4.1. Growth Curve Profiling

The growth curve profiling was obtained according to the method proposed by Vidic et al. [35]. In this method, the saturated cultures (after 24 h of incubation) were diluted in fresh LB medium (200 mL) to initial optical density (OD) of 0.05 at 600 nm and supplemented with MON’s (100 μg/mL) [18]. Samples were then incubated in a shaking incubator (200 rpm) at 37 °C [18]. The growth curves were obtained by measuring the evolution of optical density as a function of time (every hour up to 13 h) using a spectrophotometer (Shimadzu UV-2600, Tokyo, Japan) [36]. The control group was run in the same way but without MONs.

#### 2.4.2. Lethal and Sublethal Effect of TiO_2_-ZnO-MgO Mixed Oxide on Pathogenic Bacteria

Lethal and sublethal injury to the bacterial strains were assessed by serial dilution using the pour-plate method [37]. The LB medium (200 mL) was inoculated with 10 mL/L of cell suspension at 10^6^ CFU/mL (equivalent to 0.5 McFarland scale) supplemented with MONs (100 μg/mL) and were then incubated for 15 min at 37 °C (200 rpm). Then, 10 mL were placed in 90 mL of sterile peptone water (0.1%) and homogenized. Serial dilutions (up to 10^8^) were made in peptone water (9 mL), then 1 mL of diluted aliquots were plated in tryptone soy agar (TSA, DIBICO) by the pour plate method. This procedure was repeated for each treatment and each bacteria, and results were expressed as log CFU/mL. Lethality was calculated as the difference between the logarithms of colony counts in the control group without MONs and colony counts in treated samples (log No-N). To detect bacterial cell injury, dilutions of the MONs samples were pour plated in TSA supplemented with 4% of sodium chloride followed by incubation at 37 °C for 24 h [38]. The sublethal injury was calculated by the difference obtained between cultures in TSA (control group) and TSA + NaCl (after treatment) and expressed as a percentage [37,38].

Additionally, viable colonies of tested bacteria after MONs exposure were observed [39]. From the decimal dilutions (10^−4^), 0.1 mL was spread in selective medium and incubated at 37 °C for 24 h. Eosin methylene blue agar (DIFCO) was used for *E. coli*, xylose lysine deoxycholate (XLD) agar (DIFCO) for *S. paratyphi*, mannitol salt agar (DIBICO) for *S. aureus*, and blood agar (DIBICO) supplemented with 5% of human blood for *L. monocytogenes* [39].

### 2.5. Toxicity Assay

The toxicity test was performed using the early stages of *Artemia salina* [40]. *Artemia* (10 Artemia organisms in each treatment were placed into a tube with 10 mL of saline water) with 3 weeks of growth, were conserved and analyzed in saline water (25 mg/L). The TiO_2_ and MONs were suspended in 1 mL of DMSO. The *Artemia* were exposed to different concentrations (25, 50, 100 and 200 mg/mL) of treatment (Table 1) at 28 °C in darkness. *Artemia* with 1 mL of DMSO was used as a negative control. The toxicity was determined after 24 h of exposition. The number of survivors was counted, and dead *Artemia* was considered when they did not present internal or external movements during 1 min of observation. The median lethal concentration (LC_50_) was calculated using a PROBIT regression model (Software SAS System v.9.0. SAS Institute Inc., Cary, NC, USA).

### 2.6. Data Analysis

Lethal effect of MONs on *E. coli*, *S. paratyphi*, *S. aureus*, *L. monocytogenes*, and mortality on *A. salina* data were subjected to independent-samples Kruskal–Wallis non-parametric test, due to the lack of homogeneity in variances among groups (Levene’s test, *p* < 0.05) and/or normal distribution (Shapiro–Wilk W test, *p* < 0.05). A pair-wise comparison was performed using multiple comparisons of mean ranks for all groups. Furthermore, lethal concentration (LC_50_) values were subjected to one way ANOVA/Tukey test, because their variances were shown to be homogeneous (Levene´s test, *p* > 0.05) and also presented a normal distribution (Shapiro-Wilk W test, *p* > 0.05). All data were obtained from three independent experiments and each sample was performed in triplicate. Results were expressed as mean ± standard deviation. Data were analyzed using the Statistica software (v. 10 Statsoft^®^, Tulsa, OK, USA), with a significance level of α = 0.05.

## 3. Results and Discussion

### 3.1. Transmission Electron Microscopy (TEM) Studies

**Figure 1:**
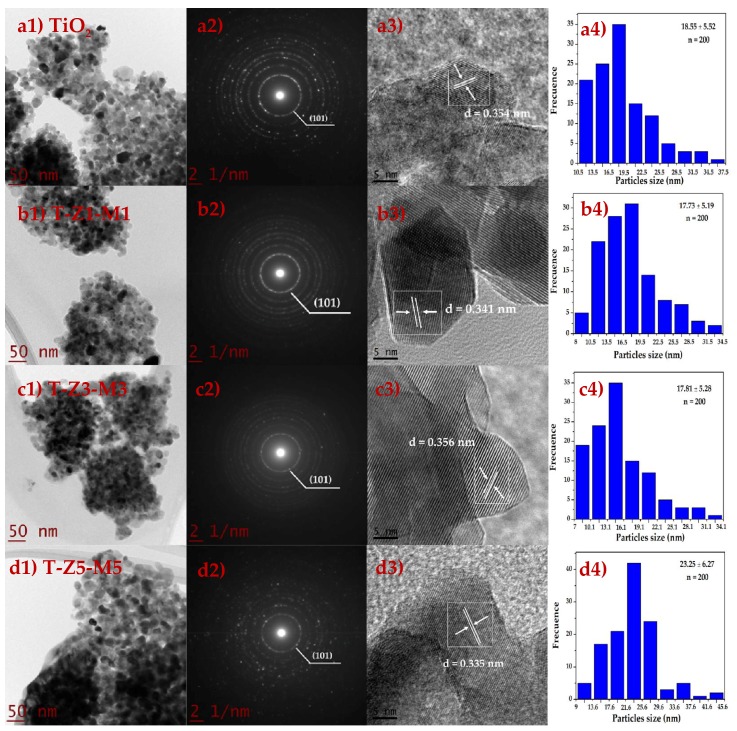
Transmission electron microscopy (**a1**,**b1**,**c1**,**d1**), selected area electron diffraction pattern (**a2**,**b3**,**c2**,**d2**), high-resolution transmission electron microscope (**a3**,**b3**,**c3**,**d3**) and the size distribution (**d1**,**d2**,**d3**,**d4**) of pure TiO_2_ and mixed oxide materials. T-Z1-M1 is TiO_2_-ZnO (1%)-MgO (1%), T-Z3-M3 is TiO_2_-ZnO (3%)-MgO (3%) and T-Z5-M5 is TiO_2_-ZnO (5%)-MgO (5%).

The morphology (TEM), lattice fringes (HRTEM), and SAED studies, as well as the mean size particles of TiO_2_ and MONs are shown in Figure 1. The corresponding TEM (Figure 1(a1,b1,c1,d1)) image shows that materials exhibited a near-spherical in shape [6,9]. Furthermore, the SAED (Figure 1(a2,b2,c2,d2)) pattern obtained for TiO_2_ and MONs and the observed ring patterns (brightness and intensity) indicating that the samples are polycrystalline in nature [9,41,42,43], in agreement with the XRD results (data published [6]). According to Maurya and Bhatia [44] discontinuous rings with spots indicate that the particles are made of rather bigger crystallites, this fact is particularly noticeable in T-Z5-M5 sample (Figure 1(d2)) [45,46]. Likewise, the lattice fringes (Figure 1(a3,b3,c3,d3)) of the TiO_2_ (0.354 nm) and MONs (T-Z1-M1: 0.341 nm; T-Z3-M3: 0.356 nm and T-Z5-M5: 0.335 nm), correspond to the TiO_2_ (101) plane (0.354 nm) of the anatase phase according to the JCPDS 21–1272 [9,46,47,48]. Moreover, the TiO_2_ and MONs exhibit mean particle size in the range 17 to 23 nm (Figure 1(a4,b4,c4,d4)). Comparable sizes and shapes were reported previously when TiO_2_ nanoparticles (15 to 50 nm) or MnO_x_/TiO_2_ nanocomposites (10–12 nm) were synthesized by the sol-gel [49], alkaline hydrothermal [47] and self-assembly [48] methods, or by modified chemical vapor condensation synthesis [50], respectively. It has been reported that the aggregation and crystallization of TiO_2_ and MONs are related to the synthesis method [51]. Other factors that may promote the agglomeration of the particles and poor crystallization during the synthesis by the sol-gel method include the temperature of calcination [52] and the concentration of precursors [53].

### 3.2. Thermal Gravimetric Analysis (TGA-DTGA) and Differential Thermal Analysis (DTA) Curves Analysis of Materials

**Figure 2:**
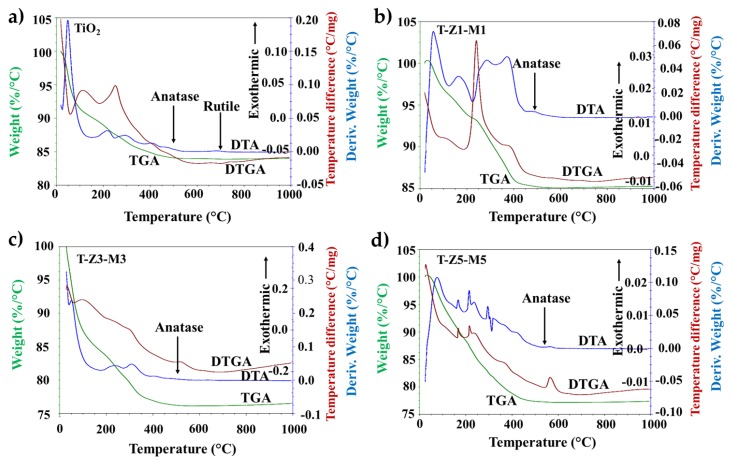
Thermal gravimetric analysis (TGA/DTGA) and differential thermal analysis (DTA) results of (**a**) TiO_2_, (**b**) TiO_2_-ZnO (1%)-MgO (1%), (**c**) TiO_2_-ZnO (3%)-MgO (3%), and (**d**) TiO_2_-ZnO (5%)-MgO (5%) mixed oxide materials.

The results of the differential thermal analysis (DTA) and the thermal gravimetric analysis (TGA/DTGA) of the TiO_2_ and MONs powders are shown in Figure 2. The TGA diagram shows three main steps for the weight loss; the first occurred in the range of 30–240 °C (weight loss approximately 15% of the total weight), corresponding to the evaporation of physically adsorbed water (30–80 °C) and to the loss of chemically adsorbed water (80–240 °C). The second, in the range of 240–500 °C (weight loss: between 4–8% for all samples), was attributed mainly to the decomposition of precursor oxyhydroxide [54]. Finally, in the range of 500–700 °C (weight loss: ≤1% in all samples), the precipitate product transformation to anatase and/or rutile takes place. Above 700 °C, the stabilization of the sample mass indicates the complete transformation of the ternary compound [55].

The endothermic effect in the derivative of thermogravimetric analysis DTGA and DTA curve at 140 °C is associated with the loss of the adsorbed water on the compound particle surface. While the peak centered at 250 and 350 °C should be attributed to the transformation of the poorly crystalline phases and the loss of structural water occluded in interplanar regions and lattice interstices [54]. On the other hand, the exothermic shoulder around 400 °C in the DTA curve corresponds to the transformation–initiation of amorphous/nano–structured compound TiO_2_–ZnO–MgO to anatase phase, whereas the exothermic peak at 430–460 °C should be attributed to the dehydroxylation/dehydration of precursors [55]. The exothermic peak around 480–500 °C in the DTA curve suggested that the transformation of the produced compound into anatase phase is completed, in accordance with the X–ray diffraction results reported previously [6]. At around 700 °C in the DTGA and DTA curve the rutile phase start to appear in pure TiO_2_ sample. Thereafter no significant thermal effects can be detected even at a temperature as high as 1000 °C. The thermal stability of the MONs may be attributed to the presence of MgO since the MgO plays an important structural role when composites are synthesized [56].

### 3.3. Growth Curve Profiling

**Figure 3:**
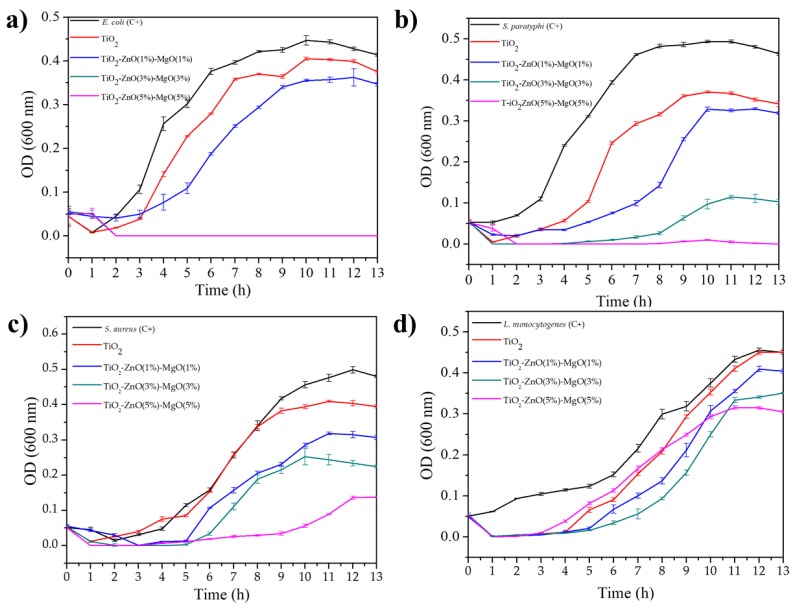
Growth curves profiling of *E. coli* (**a**), *S. paratyphi* (**b**), *S. aureus* (**c**) and *L. monocytogenes* (**d**) exposed to TiO_2_ and mixed oxide nanoparticles.

Figure 3 shows the kinetic effect of the TiO_2_ and MONs on *E. coli* (Figure 3a), *S. paratyphi* (Figure 3b), *S. aureus* (Figure 3c) and *L. monocytogenes* (Figure 3d). It is clear that under the standard conditions (C+) bacterial cells were quickly adapted to the medium and reached their exponential phase normally (in all bacterial tested). However, this normal behavior was affected by the presence of MONs, particularly in TiO_2_–ZnO (3%)–MgO (3%) and TiO_2_–ZnO (5%)–MgO (5%) treatments, which showed an increasing lag phase and a decreasing log phase [17,57]. Also, TiO_2_–ZnO (3%)–MgO (3%) and TiO_2_–ZnO (5%)–MgO (5%) treatments exhibited the same behavior on *E. coli* growth (Figure 3a). These results suggest that MONs exhibited reduced kinetics of antibacterial activity, which were in agreement with the results of Venkatassubbu et al. [18] when TiO_2_ (100 µg/mL) and ZnO (100 µg/mL) nanoparticles were evaluated *on Salmonella typhi*, *Klebsiella pneumonae*, and *Shigella flexneri*. The authors highlighted a bacteriostatic effect of TiO_2_ and ZnO against Gram–negative bacteria. In general, *S. aureus* (Figure 3c) and *L. monocytogenes* (Figure 3d) exhibited fewer inhibition rates than *E. coli* (Figure 3a) and *S. paratyphi* (Figure 3b) to the TiO_2_ and MONs [6]. Freire et al. [57] reported that the ternary colloidal–system chitosan–silver–fluoride nanocomposite was effective in inhibiting the growths of *P. aeruginosa* (67.5%) *S. aureus* (40%) and *E. coli* (57%). In addition, similar trends were previously reported by Li et al. [16] when evaluated the antimicrobial effect of Ag–TiO_2_–Chitosan on *E. coli* (Gram-negative) and *P. aeruginosa* (Gram-positive), and suggested that the antimicrobial effect might be related to the species of bacteria and attributable by the variation on their cell enveloped (superficial electrostatic charges and cell physiology–morphology). It has been reported that bacteria could change the electrostatic charges on the superficial molecules by the expression of some genic products that promote the addition of amino acids (e.g., Lysil–phosphatidylglycerol) in their structure, which may provide a protective effect against cationic compounds [58]. Therefore, the antimicrobial effect of the MONs against bacteria could decrease due to this natural mechanism, but these points has not been studied yet. Hassan et al. [17] evaluated the toxicity of Ce_2_O_3_/TiO_2_ composite nanofibers against *S. aureus* and *S. Typhimurium* and they reported that the antibacterial effect may also be related to the type of doping–material. Furthermore, these authors reported that morphologies of both strains were deformed by effect of Ce_2_O_3_/TiO_2_ nanocomposite, which may be caused by the direct contact or electrostatic interaction of nanocomposites with the cell surface and the production of oxidant species as HO^•–^, O_2_^•2–^, HO^2•–^ and H_2_O_2_ produced by the interaction of TiO_2_ with the medium [17,18]. However, the exact antimicrobial mechanism of TiO_2_ nanoparticles (alone or combined) is still unknown [49]. It has also been reported that nanoparticle morphology and high surface are other factors to increase the antimicrobial activity against pathogenic bacteria [59].

### 3.4. Lethal and Sublethal Damage of Mixed Oxide Nanoparticles (MON’s) on Pathogenic Bacteria

Table 1 shows that all treatments caused a reduction in the plate count (*p* < 0.05) in all tested bacteria (initial cell concentration was approximately 6.6–6.8 log CFU/mL). The lowest reduction ranges were obtained with TiO_2_ (0.58–0.95 log CFU) and TiO_2_–ZnO (1%)–MgO (1%) (0.71–0.96 log CFU) treatments, while the highest reduction values were obtained applying TiO_2_–ZnO (3%)–MgO (3%) (1.67–2.05 log CFU) and TiO_2_–ZnO (5%)–MgO (5%) (1.63–2.10 log CFU) treatments. It has been reported that pure TiO_2_ exhibited a poor reduction on *E. coli* (8.7%), but its antibacterial effect may be improved (reduction >80%) by the presence of other compounds in the TiO_2_–matrix as it was demonstrated by He et al. [60] or by Dhanalakshmi et al. [41] who reported major antimicrobial activity of ZnO–TiO_2_ nanocomposite against *E. coli* and *Bacillus cereus* compared to the obtained with pure TiO_2_. Nonetheless, similar lethal values were observed in the last two treatments (T–Z3–M3 and T–Z5–M5) on all tested bacteria. Similar trends were reported by Yamato et al. [61] who observed changes in antibacterial activity when ZnO–MgO nanocomposite was evaluated at different doping amounts of ZnO (1:0, 8:2, 6:4, 4:6) on *E. coli* and *S. aureus*. Furthermore, the authors highlighted that an excess of doping material on nanocomposite may affect antimicrobial activity. In this case, a saturation of ZnO–MgO on TiO_2_–matrix in T–Z5–M5 treatment may influence its antimicrobial activity or any potential application [3].

**Figure 4:**
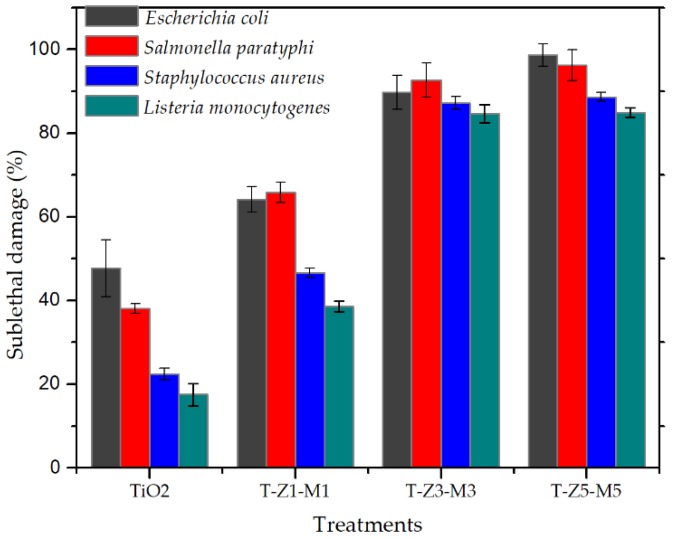
Sublethal damage of TiO_2_ and mixed oxide nanoparticles (MONs) on pathogenic bacteria. T–Z1–M1 is TiO_2_–ZnO (1%)–MgO (1%), T–Z3–M3 is TiO_2_–ZnO (3%)–MgO (3%) and T–Z5–M5 is TiO_2_–ZnO (5%)–MgO (5%).

Sub–lethal injury is related to the high sensitivity of bacterial cells to stress conditions after any treatment and the ability of cells to survive at adverse conditions [38,62]. To the best of our knowledge, the sublethal injury test is commonly used to evaluate the effect of any food–preservation treatment on food–borne pathogens and their ability to survive external conditions [63,64]. Sublethal damage ranged from 17 to 98% but it was dependent on each bacteria and each treatment as shown in Figure 4. According to García et al. [37], a killer treatment should exhibit ≥99% of sub–lethal cell damage. In this context, a bacteriostatic effect by the MONs is evidenced in accordance with those results previously reported by Venkatassubbu et al. [18] using TiO_2_ nanoparticles at 100 µg/mL on Gram–negative bacteria. The gram–negative bacteria were more sensible than the gram–positive bacteria to the MONs [6]. Nonetheless, the sublethal injury of tested bacteria could be related to the composition of each evaluated composite, and to the bacteria cell wall composition [17,59], but also their ability to form biofilm as protection mechanism [65]. It has been reported that under hostile external conditions, the cell will try to survive and repair by themselves by natural mechanisms [62]; however, if the adverse conditions are extended, they may result in cellular death [66]. In our study, the TiO_2_ and MONs exhibited a bacteriostatic effect and bacterial cells may be repaired and reproduced under these treatments.

**Figure 5:**
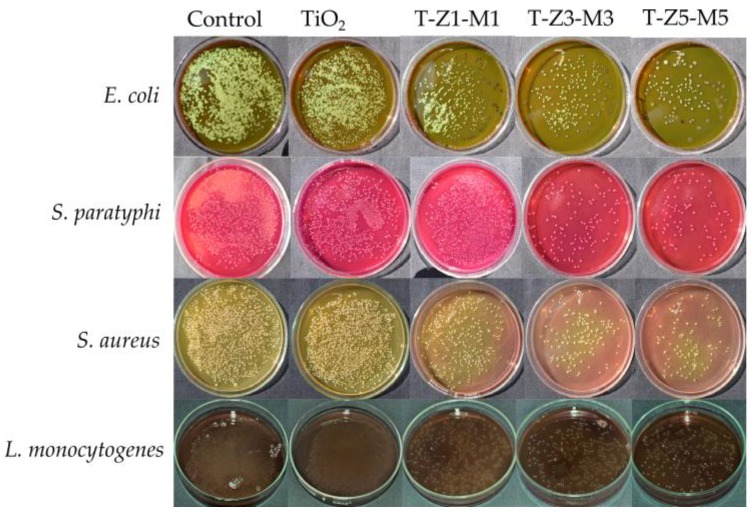
Viable cells of different pathogenic bacteria under TiO_2_ and mixed oxide nanoparticles. T–Z1–M1 is TiO_2_–ZnO (1%)–MgO (1%), T–Z3–M3 is TiO_2_–ZnO (3%)–MgO (3%) and T–Z5–M5 is TiO–-ZnO (5%)–MgO (5%).

Figure 5 shows the viable cells of different pathogenic bacteria under TiO_2_ and MONs. As can be seen under favorable conditions bacterial cells can survive and reproduce [37]. In contrast, Masae et al. [39] reported no viable cells of *E. coli* after 15 min of exposure to Se–doped TiO_2_ nanoparticles (applying fluorescent light and a concentration of 500 mg of powder/L) compared with the control (TiO_2_).

Differences in results may be due to the concentration of powder employed (100 mg/L compared to 500 mg/L) without considering the exposition to the fluorescent light, which may enhance the antibacterial activity of MONs by the photocatalytic phenomenon [54,60]. Further studies are recommended by using visible light and investigating their effect on photocatalytic activity of MONs on pathogenic bacteria.

### 3.5. Mortality (%) of Artemia Salina and Toxic Effect (LC_50_) of Mixed Oxide Nanoparticles (MONs)

*A. salina* is an important model used for the preliminary assessment of general toxicity of inorganic nanoparticles [21,24,25]. The mortality (%) values of *A. salina* in the presence of TiO_2_ and MONs (17 to 23 nm) are summarized in Table 2. The adult *A. salina* (without nanoparticles) did not exhibit any alteration, such as low motility or cannibalism in their behavior, and similar rates of mortality to the controls were observed at a concentration of 25 mg/L (except TiO_2_ with 3.4% of mortality). For instance, mortality increased from 3 to 13% at 50 mg/L, while at 100 mg/L it increased from 10 to 20%, and the highest rate of mortality was observed in MONs at 200 mg/L (30 to 50%), but less than by pure TiO_2_ (73.34%). Khoshnood et al. [67] reported mortality rates of 6.7, 16.67 and 46.67% on *Artemia franciscana* (after 24 h of exposure) in the presence of TiO_2_ nanoparticles (~20 nm) at concentrations of 20, 50 and 100 mg/L, respectively. Ates et al. [24] reported a mortality rate of 3 to 5% of *A. salina* in the adult state after exposure for 24 h to TiO_2_ (100 mg/L) nanoparticles (>200 nm), and no differences were found in nauplii and adult in the same study. Our results are in agreement with those reported in *D. magna* (100 mg/L of TiO_2_ with 13% of mortality after 24 h of exposure) [68]. Differences in results may be attributable to the size (12 to 40 nm) of nanoparticles as mentioned by Kim et al. [69]. In addition, it was reported that *A. salina* in the adult state is less susceptible to metallic compounds compared to the young-larvae *Artemia* (≤5 days of life), because a functional digestive system is not present in the younger states of the crustacean [70]. On the other hand, it has been reported that the mortality of *A. salina* in the presence of any compound increased significantly with increasing concentration and time of exposure [33].

The toxicity values (LC_50_) of TiO_2_ and MONs on *A. salina* are given in Table 3. TiO_2_ exhibited an LC_50_ value of 140 mg/L. These findings are in accordance with those of Ates et al. [24], who reported that aqueous suspension of TiO_2_ (LC_50_ value >100 mg/L) nanoparticles were not acutely toxic to *A. salina* at a concentration of 100 mg/L. Similar results were observed by Wiench et al. [68] who reported low acute toxicity of TiO_2_ (nano and micro scale) on *D. magna* (LC_50_ >100 mg/L). The T–Z1–M1 (238 mg/L), T–Z3–M3 (891 mg/L) and T–Z5–M5 (1468 mg/L) treatments exhibited an increase in their LC_50_ values with significant differences (*p* < 0.05) compared to TiO_2_ (144 mg/L). This behavior suggested that the presence of ZnO and MgO into the TiO_2_ matrix decreased the toxicity of pure TiO_2_ [67,68]. Khoshnood et al. [67] reported that the toxicity pattern (individually) of metal oxides to *A. franciscana* was TiO_2_ > ZnO, while in *Artemia* sp. the pattern toxicity of inorganic nanoparticles was Ag > CuO > ZnO > Au > TiO_2_ > SiO_2_ [32]. Conversely, Ozkan et al. [33] reported that Ag-TiO_2_ (43 nm) nanocomposite (LC50 = 23 mg/L) was found to be up to 17-fold more toxic than pure TiO_2_ (44 nm) nanoparticles (LC50 = 381 mg/L) on *A. salina*.

The toxicity status of the TiO_2_ and MONs were classified using the Clarkson´s toxicity index as follows: toxic (LC_50_ of <100 mg/L), medium toxic (LC_50_ of 100–500 mg/L), low toxic (LC_50_ of 500–1000 mg/L) and non-toxic (LC_50_ >1000 mg/L) [71]. In this context, the TiO_2_ and T–Z1–M1 treatments showed medium toxicity, while T–Z3–M3 and T–Z5–M5 exhibited a low and non-toxic effect, respectively, on *A. salina*. Earlier studies have reported non-toxic effects of nano–TiO_2_ on *A. salina*, *A. franciscana*, *D. magna* and *Danio rerio* embryos at high concentrations (100–500 mg/L) [24,26,34,72,73,74].

It has been reported that the toxic effect of TiO_2_ on biological models may be related to the photo-catalytic (photo-activation) behavior of TiO_2_, and ROS production resulting in oxidative stress on the organisms [27]. Ma et al. [30] investigated the effect of TiO_2_ on *D. magna* under UV–radiation (UVR) and reported that the LC_50_ values under UVR was 29.8 mg/L, and was 500 mg/L without UVR. The authors also indicated that TiO_2_ toxicity under simulated solar radiation (1700 µm cm^−2^ s^−1^, which correspond to 25% of natural solar radiation on a sunny day) decreased compared to the toxicity under UV–B and UV–C exposure. Hund-Rinke and Simon [29] proposed that the toxic effects of TiO_2_ on crustacean models may be decreased due to the intake of nanoparticles by the organism reducing the capacity of TiO_2_ to interact with water molecules and UVR to produce ROS [63]. In addition, the activation of the antioxidant system of an organism may be achieved as suggested by Liu et al. [73]. It has also been demonstrated that the bioaccumulation of nanoparticles inside the gut of *A. salina* does not induce mortality after 24 h of exposure [74]. UVR was not applied in our study, and therefore further studies are needed with other biological models to evaluate the possible toxic effect of MONs.

Additionally, it must be emphasized, that increasing ZnO and MgO concentrations into MONs (in particular for T–Z3–M3 and T–Z5–M5), a moderate increase in antibacterial features against *E. coli*, *S. paratyphi*, *S. aureus*, and *L. monocytogenes* and a significant decrease in toxicity on *A. salina* compared to the pure TiO_2_ were observed. These behaviors may be attributable to the physiology of each studied biological model and their sensitivity or response to the MONs, in particular, by the differences between the prokaryotic and eukaryotic cell structures [75]. For example, the plasmatic membranes of eukaryotic cells are more complex in their phospholipid profile than the prokaryotic cells [76], limiting the interaction between MONs and superficial molecules of eukaryotic cells. Moreover, MONs did not cause toxicity in *A. salina* within the range of antimicrobial concentrations; evidencing that the tolerance of *A. salina* (pluricellular model) to the MONs is higher than the bacteria (unicellular model) [76,77]. Thus, the low toxicity observed in *A. salina* can be explained by the ability of eukaryotic models to eliminate or excrete (possible chelating metals mechanism) the MONs, by their capacity to neutralize the generated ROS species from TiO_2_ and MONs, by activation of the antioxidant system mechanism, and by their cellular regeneration capacity in comparison with the bacterial cells [29,73,74,77,78]. In consequence, the negative effects of MONs on the eukaryotic cell were diminished. However, it must be considered that the LC_50_ test on *A. salina* is not a predictor of antibacterial activity [79]; although LC_50_ values on *A. salina* test have a good correlation with the obtained in mice (*r* = 0.85; *p* < 0.05) for the same plant extract [74]. Freire et al. [57] reported a good antimicrobial activity of colloidal chitosan-silver-fluoride nanocomposite against *S. aureus*, *E. coli*, *Enterococcus faecalis*, *P. aeruginosa*, and *Candida albicans*, with an LC_50_ value >1000 mg/mL classifying as low toxicity in *A. salina*, and suggested that the use of colloidal nanocomposite presented no substantial risk to human health. Furthermore, Shriniwas and Subhash [80] evaluated the antibacterial activity and toxic effects of silver nanoparticles on *A. salina* and reported good to moderate antibacterial activity against *S. aureus*, *E. coli*, and *P. aeruginosa* and without toxic effects on *A. salina* (LC_50_ of 515 mg/L). Conversely, Kumar et al. [81] reported a good antimicrobial activity of silver nanoparticles (10–100 nM) against five clinical pathogenic bacteria (*E. coli*, *Klebsiella pneumoniae*, *S. typhii*, *S. aureus* and *Vibrio cholerae*), however, the 50% of *A. salina* pollution mortality was observed at low concentrations (10 nM/mL) of nanoparticles, evidencing a cytotoxic effects (LC_50_ < 100 mg/L) of this material on the crustacean model. According to Ullah et al. [82] and Clarkson et al. [71] an LC_50_ value of <100 mg/L of any compound (organic and/or inorganic) on *A. salina* is considerable as toxic for human consumption, however, compounds with LC_50_ < 100 mg/L could exhibit potential activity as chemoprotective agents [83].

## 4. Conclusions

TiO_2_-ZnO-MgO mixed oxides nanomaterials (MONs) based TiO_2_ have a good thermal stability. MONs presented a bacteriostatic effect on Gram-negative and Gram-positive bacteria, which were strongly influenced by the type of microorganism and MONs composition, where TiO_2_–ZnO (5%)–MgO (5) presented the highest inhibition rate on all bacteria tested. Furthermore, MONs have medium (TiO_2_ and TiO_2_–ZnO (1%)–MgO (1%)), low (TiO_2_–ZnO (3%)–MgO (3%)) and non-toxic (TiO_2_–ZnO (5%)–MgO (5%)) effects on *A. salina*, although it was dependent on each treatment. Further studies are needed to evaluate the potential industrial applications of MONs based TiO_2_.

## Figures and Tables

**Table 1:** Lethal effect of TiO_2_ and mixed oxide nanomaterials on pathogenic bacteria.

Treatment		Reduction Log CFU			
	Code	*E. coli*	*S. paratyphi*	*S. aureus*	*L. monocytogenes*
TiO_2_	-	0.95 ± 0.01 b	0.66 ± 0.01 c	0.62 ± 0.04 c	0.58 ± 0.01 c
TiO_2_–ZnO (1%)–MgO (1%)	T–Z1–M1	0.96 ± 0.07 b	0.90 ± 0.07 b	0.75 ± 0.02 b	0.71 ± 0.01 b
TiO_2_–ZnO (3%)–MgO (3%)	T–Z3–M3	2.05 ± 0.06 a	1.94 ± 0.02 a	1.76 ± 0.01 a	1.67 ± 0.02 a
TiO_2_–ZnO (5%)–MgO (5%)	T–Z5–M5	2.10 ± 0.07 a	1.87 ± 0.02 a	1.72 ± 0.01 a	1.63 ± 0.02 a

Values are the average ± standard deviation (*n* = 9). Different letters in each column indicate significant statistical differences between treatments (α = 0.05).

**Table 2:** Mortality values (%) of *Artemia salina* at different concentrations of TiO_2_ and MONs.

Concentration (mg/L)	Treatment/Mortality (%)			
	TiO_2_	T-Z1-M1	T-Z3-M3	T-Z5-M5
0	0	0	0	0
25	3.33 ± 0.57 d	0	0	0
50	13.34 ± 0.57 c	6.67 ± 0.57 c	3.34 ± 0.57 c	0
100	20.00 ± 1.00 b	26.66 ± 0.57 b	16.34 ± 0.57 b	10.00 ± 0.01 b
200	73.34 ± 0.57 a	50.00 ± 0.01 a	30.00 ± 0.01 a	30.00 ± 0.01 a

Values are the average ± standard deviation (*n* = 30). Different letters in each column indicate significant statistical differences between concentrations (α = 0.05). T–Z1–M1 is TiO_2_–ZnO (1%)–MgO (1%); T–Z3–M3 is TiO_2_–ZnO (3%)–MgO (3%), and T–Z5–M5 is TiO_2_–ZnO (5%)–MgO (5%).

**Table 3:** Lethal concentration (LC_50_) values and toxicity index (Clarkson´s index) of TiO_2_ and MONs on *A. salina*.

Treatment	Lethal Concentration (mg/L)	Clarkson’s Toxicity Index	
		Range (mg/L)	Results
TiO_2_	140.36 ± 11.90 d	<100, toxic	Medium toxicity
T–Z1–M1	238.08 ± 29.62 c	100–500, medium toxic	Medium toxicity
T–Z3–M3	891.11 ± 42.26 b	500–1000, low toxic	Low toxicity
T–Z5–M5	1468.18 ± 10.05 a	>1000, non-toxic	Non-toxicity

Values are the average ± standard deviation (*n* = 30). Different letters in each column indicate significant statistical differences between treatments (α = 0.05). T–Z1–M1 is TiO_2_–ZnO (1%)–MgO (1%); T–Z3–M3 is TiO_2_–ZnO (3%)–MgO (3%), and T–Z5–M5 is TiO_2_–ZnO (5%)–MgO (5%).
